# Nutritional quality of lunch meals and plate waste in school lunch programme in Southern Thailand

**DOI:** 10.1017/jns.2022.31

**Published:** 2022-05-12

**Authors:** Jaruneth Petchoo, Narisara Kaewchutima, Nattapol Tangsuphoom

**Affiliations:** 1 Department of Community Public Health, School of Public Health, Walailak University, Tha Sala, Nakhon Si Thammarat 80160, Thailand; 2 Food Technology and Innovation Research Center of Excellence, Walailak University, Tha Sala, Nakhon Si Thammarat 80160, Thailand; 3 Department of Environmental Health, School of Public Health, Walailak University, Tha Sala, Nakhon Si Thammarat 80160, Thailand; 4 Food and Nutrition Academic and Research Cluster, Institute of Nutrition, Mahidol University, Salaya, Nakhon Pathom 73170, Thailand

**Keywords:** Nutritional quality, Plate waste, School lunch, School lunch programme, Waste quantification

## Abstract

Food waste globally affects food security and sustainability. There currently are few studies focusing on food waste in schools. The present study aimed to determine the meal quality and plate waste in school lunch programme in Thailand. This cross-sectional study was conducted in canteens of representing kindergarten and elementary schools in Southern Thailand within their real-life context. The assessment was carried out over five consecutive school days in July–August 2020 for each school, at which two types of menus, including rice with side dish and one-dish meal were served. Waste collection and quantification were conducted by selective aggregate weighing, according to the Food Loss and Waste Protocol. All lunch menus contained excess rice but insufficient meats, fruits, vegetables and eggs, regarding the national lunch standard. For each serving portion, 7–33 % rice, 9–22 % meats, 7–65 % vegetables, 1–19 % fruits and 3–14 % eggs were discarded. Plate waste from rice with side dish menus (10–29 %) was more than one-dish meals (7–17 %). By estimation, each kindergartener generated 71⋅4 g plate waste daily, accounting for the caloric and monetary values of 146 kcal and 0⋅16 US dollar. The amount of plate waste and the respective caloric and monetary losses were less obvious at the elementary school. Plate waste affected the adequacy of food intakes of schoolchildren from lunch meals. The data obtained from the present study will be useful for planning and implementation of school lunch programme in Thailand and countries with similar context.

## Graphical abstract

**Figure:**
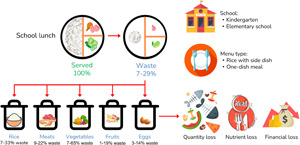

## Introduction

Malnutrition in pre-school and schoolchildren becomes an important public health issue in many developing countries, including Thailand. According to the 5th National Health Examination Survey in 2014, about 400 000 (3⋅5 %) Thai children aged 1–14 years were stunted, while another 470 000 (4⋅1 %) children were underweight^(1)^. On the other hand, the prevalence of overnutrition in schoolchildren had continuously increased, as 670 000 (5⋅9 %) and 790 000 (7⋅0 %) children were overweight and obese, respectively. Apart from macronutrient imbalance, >50 % of children in Thailand had insufficient intake of micronutrients, particularly vitamin A, vitamin D, iron, iodine and calcium. Such imbalance and deficiencies cause various health problems to children^(2)^. Data from the Southeast Asia Nutrition Survey in 2013 revealed that 45–53 % of children in Southeast Asian countries were malnourished, and those stunted children were more likely to have Intelligence Quotient below the global average^(3)^. The retarded growth, as well as impaired physical and cognitive development, adversely affect the children's quality of life, not only in their childhood, but also evident throughout the life cycle. Therefore, in many countries, child nutrition has been addressed as a national programme, for which agencies in public sector work together to stipulate the measures to tackle nutrition problems in the child population^(2)^.

School feeding programme have been successfully implemented to improve nutritional status, to provide nutrition education, as well as to promote healthy development and growth of children in developed countries like USA, Finland and Japan. The coverage of such programme varies in each country, depending on the economic status, nutritional and health problems, and dietary patterns^(4–6)^. In Thailand, to ensure that schoolchildren receive nutrients based on their nutritional needs, the government has provided lunch and one serving of milk on every school day for each student nationwide since 1993^(7)^. Presently, the budget of 20 Thai baht (0⋅65 US dollar) per student per day is allocated to each public and private school to acquire proper lunch for their students. At schools, meal planning is conducted by the responsible teacher who, in most cases, has limited background in nutrition. This might result in lunch meals with imbalanced macronutrients or insufficient micronutrients to fulfil nutritional requirement of the students^(8)^. In assuring that the provided meals contain adequate amount of nutrients, the Lunch Standard for Thai Children has been developed and adopted as a guideline for meal arrangement in school lunch programme. This guideline provides recommended amount and frequency of different food groups for weekly planning of school lunch menu for children at various ages, as well as the amount of raw ingredients needed for meal preparation^(9)^. Since 2013, an automated online platform, namely Thai School Lunch, has been launched to ease the school in arranging lunch menus according to the recommendation and the budget. The platform also provides the nutrient content of selected menus, in addition to the type, amount and budget needed for ingredients. It helps the schools to assess the nutritional values and cost of the arranged menu on a real-time basis, which provides flexibility and convenience in menu planning of school feeding programme^(10)^.

Presently, food loss and waste, which refers to food that fits for human consumption but being discarded from the food supply chain, has become a global issue. Approximately one-third of the world's produced food was discarded, resulting in the disposal of around 1⋅6 billion tons of food each year^(11)^. Such an issue has been addressed as one of the United Nations’ Sustainable Development Goals to cut down half of the amount by 2030^(12)^. It is revealed that in high-income countries, a large amount of food is thrown away by the consumers at the consumption stage of the food supply chain^(13)^. Plate waste, which is the served food that is discarded or leftover in catering service such as hotel restaurants, dining commons and canteens, has been extensively studied to identify the root causes and efficient reduction strategies^(14,15)^. The assessment of plate waste has been also performed in hospital wards, and senior homes as an indirect measurement of food intake of individuals^(16)^.

Many previous studies have demonstrated that portion size, serving style and practices, as well as preference on menu, affected the amount of plate waste generated in food services of academic institutions. School food waste in many cases arose from the lack of attention to students’ dietary habits and low self-efficacy of students to finish their meals^(17,18)^. Other factors affecting plate waste generation included menus, meal pairings, size of serving bowls, food logistics and distance to the dining hall^(4,19,20)^. A study conducted in China indicated that the major cause of food waste from school food services was students did not finish their meals. Moreover, the quality and efficiency of catering service, student dietary preferences and food culture also affected the amount of food waste generated in school canteen^(21)^. Consequently, when the students could not finish the entire portion, their actual nutrient intake from lunch might deviate from what intended to provide through lunch meals. This would affect the efficacy of school lunch programme in improving nutritional status, as well as growth and development of schoolchildren^(22)^.

Although there are previous studies on quantification of food waste in school canteens, they were not undertaken in Thailand, particularly in school lunch programme^(13,14,16)^. Moreover, culinary culture, eating habits, and meal serving practices in European countries, where most of the studies were conducted, are different from those in Asian countries^(23)^. Therefore, the present study aimed to assess the nutritional quality of lunch meals and the amount and composition of plate waste in school lunch programme in Thailand. The nutritional and financial impacts of such plate waste were also determined.

## Methods

### Study design and study site selection

This cross-sectional study was conducted in two public schools in Thung Song district of Nakhon Si Thammarat province, Thailand (Latitude: 8°9′31⋅1436″N; Longitude: 99°40′26⋅3568″E). Nakhon Si Thammarat is the most populated province in the southern part of Thailand, in which 16⋅5 % of the population are residing, and Thung Song is the second most populated district of the province^(24)^. Budget for school lunch programme is allocated by governing agencies overseeing the school, including six public schools under the Local Administrative Organization, while other forty-five public and fourteen private schools are governed by the Ministry of Education^(25)^. We purposively selected two schools under the Local Administrative Organization, both of which have implemented the school lunch programme. For these schools, it is more feasible and flexible to implement campaigns on awareness raising and reduction of plate waste in the future. The inclusion criteria for study sites were: (1) menu planning is performed using Thai School Lunch platform; (2) food is prepared and cooked on-site and (3) school management have consent and commitment to participate. This study was conducted according to the guidelines laid down in the Declaration of Helsinki, and all procedures involving human subjects were approved by the Human Research Ethics Committee of Walailak University (Approval Number: WUEC-19-173-01). Informed written consent was obtained from and signed by all teachers and school staff involved in feeding programme. The study purpose, procedures, possible risks and benefits were explained to participants in local languages. Confidentiality of information collected from each study participant was kept. They were informed that they have the full right to withdraw from the study at any time if they face any difficulties.

The study sites consisted of a kindergarten school with 531 students of K1–K3 (aged 4–6 years), and an elementary school with 239 students of Year 1–Year 6 (aged 7–12 years). Anthropometric data of pupils in both schools, given in Supplementary Table S1, was acquired from the measurements performed by teachers, as a part of student's annual physical examination of the Ministry of Public Health. Anthropometric status was assessed as weight-for-age, height-for-age and weight-for-height, using cut-off points and classifications of the World Health Organization^(26)^, and the Bureau of Nutrition, Department of Health, Ministry of Public Health^(27)^.

### School lunch programme and school food environment

Each school has a teacher in charge of school lunch programme who performs menu planning, budget management and procurement of foodstuffs. Menu planning was conducted using Thai School Lunch platform to ensure that the provided lunch met nutrient requirements of the students within budget. The menus were set for a 2-week (10 school days) cycle ahead of time throughout the school year. Preparation and cooking were performed daily by a school cook who also helped the teacher in serving the food. Each student received a portion of lunch consisting of a main dish, and a portion of dessert or fresh-cut fruit. Main dishes were either steamed rice with side dish, such as stir-fried meat and vegetable, meat and vegetable soup, or one-dish meal, such as fried rice and noodle soup. In each week, rice with side dish menus were served for 3 d and one-dish meals were alternately served on another 2 d. For both schools, students did not bring their own lunch to school. So, the provided school lunch was their only food choice at lunch. There was not any food and drink vendor or vending machine in the canteen and within the school enclosure. Although there were food stalls and restaurants located within walking distance in the school neighbourhood, students were not allowed to leave the school gate until the end of school day.

### Lunch meal and plate waste assessment

Assessment was carried out in each school within its real-life context over five consecutive normal school days in July–August 2020. Before the lunch time on each assessment day, ten portions of the served food were randomly drawn from the serving line^(22)^. Weights of each portion, and of each food item in each portion, were determined using a digital balance (Kitchen Scale KJ-114, Tanita Corporation, Tokyo, Japan) to the nearest 0⋅1 g. We categorised food items into five groups based on the ingredients of the served menus as listed in the Lunch Standard for Thai Children, including rice, fruits, vegetables, meats and eggs. Such food groups were in consistent with the food categories of the Food and Agriculture Organization of the United Nations^(28)^.

Waste collection and quantification were conducted by selective aggregate weighing, according to the Food Loss and Waste Protocol of the United Nations Environment Programme^(28)^. We defined food waste as the food left uneaten after lunch, excluding inedible parts such as animal bones and fruit peels. Liquid part of soup and curry was excluded from the assessment. A recording station equipped with a digital scale and waste collection containers was set up in the school canteen.

On each assessment day, collection containers including trash bags and plastic buckets were labelled and colour-coded for each food group. Lunches were served in usual manner of each school and the students were allowed to have their lunches without disturbing, rushing or forcing to finish. About 20 % of the students (108 kindergartners and 48 elementary students) were randomly asked by the researchers to surrender their plates after finish eating, upon their verbal consent and willingness to participate in the study. After draining off the liquid part and removing inedible items, the leftover food was scraped into separate collection containers of five food groups, as mentioned earlier. At the end of each assessment day, the collected waste of each food group was weighed separately prior to pooling together. A sample of 200 g for each pooled sample was drawn and kept frozen for further nutrient analysis. The remainder of food scrap was placed in the school dumpster until being collected by the municipal. At both schools, food waste is discarded together with other garbage without sorting or any other waste management. The waste is collected on every other day by the municipal and manually sorted. Recyclable garbage is sold to the recycling factory while organic waste is being composted and landfilled.

### Data analysis

#### Composition and nutrients of lunch meals

Serving portion and composition of lunch meals were obtained from the total weight and the weight of each food group presented in ten portions of each menu. Nutritional composition of lunch meals, including carbohydrate, protein, fat and energy of each serving portion was calculated from its composition using INMUCAL-Nutrients V.4.0 software (Institute of Nutrition, Mahidol University, Nakhon Pathom, Thailand) and the data available from Online Thai Food Composition Database 2015^(29)^. The amounts of each food group and nutrient of the provided lunch were also calculated as the percentage of those of Lunch Standard for Thai Children^(9)^, according to the respective age group of the students (3–5 years for kindergarten, and 6–12 years for elementary), as follow:
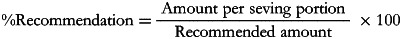


#### Quantity and composition of plate waste

The total quantity of food scraps and quantity of each food group collected on each assessment day was expressed as the amount per capita by dividing with the number of portions sampled at each school (108 plates for kindergarten and 48 plates for elementary school). The percentage of wasted food was calculated according to the following formula:
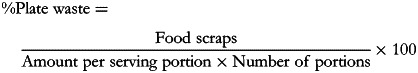


#### Macronutrients of plate waste

Samples of plate waste were analysed for proximate composition by the Laboratory Testing Service of the Central Laboratory (Thailand) Co., Ltd. in Songkhla, Thailand. In total, there were five samples for each school, including the scraps of three rice with side dish and two one-dish meals. Analyses of moisture, crude protein (Nx6.25), crude fat and ash were performed in duplicates, according to the AOAC Official Methods^(30)^. Total carbohydrate content was calculated by subtracting the percentage of moisture, crude protein, crude fat and ash from 100. Energy was calculated by using the caloric content of macronutrients which were 4 kcal/g for protein and carbohydrate and 9 kcal/g for fat. Data were reported as nutrient and calorie content per 100 g of plate waste.

#### Impacts of plate waste

Actual intake for each food group from lunch meals was estimated by subtracting the amount in the served meals with that contained in plate waste. The percentage of the consumed amount of each food group to the recommendation was also calculated as described earlier. For each school, the quantity loss per capita was calculated by averaging the quantity of plate waste over the 5-d assessment period. Caloric loss per capita was obtained from the caloric content of the plate waste. The cost of plate waste per capita was estimated by multiplying the percentage of food waste per capita with daily budget allocated for school lunch programme, i.e., 20 Thai baht (0⋅65 US dollar) per student. Impacts of plate waste generated in each school were also expressed as school day and school year basis. Daily impact was calculated by multiplying the number of students in each school (531 for kindergarten and 239 for elementary school) with the per capita values of quantity, calories and cost of plate waste. Yearly impact over the school year (200 school days) was also calculated.

### Statistical analysis

The data were analysed using the statistical software IBM^®^ SPSS^®^ Statistics Version 20 (IBM, Armonk, New York, USA). Results were presented as means and standard deviations. Statistical differences were determined at the *P*-value of <0⋅05 using the Mann–Whitney *U* test.

## Results

### Subject characteristics

The ratios between numbers of boy and girl students in both schools were similar at about 1:1 (Supplementary Table S1). The majority of pupils (about 82 and 70 % for kindergarten and elementary school, respectively) had normal weight-for-height. For both schools, less than 10 % of the students were underweight but the ratio of overweighed students was doubled for the elementary school. Regarding the family and socioeconomic background of the students, about half of the parents aged 41–50 years (41⋅3 %) and attained high school education (43⋅3 %). Their families were single households with 3–4 members. The average household annual income was approximately 324 000 Thai baht (10 800 US dollars), which is comparable to that of the households in the vicinity of Bangkok – the capital city of Thailand. It has been reported that those families spend about 6500 Thai baht (220 US dollars) for their monthly food purchase^(31)^.

### Composition and nutrients of lunch meals

**Fig. 1.**
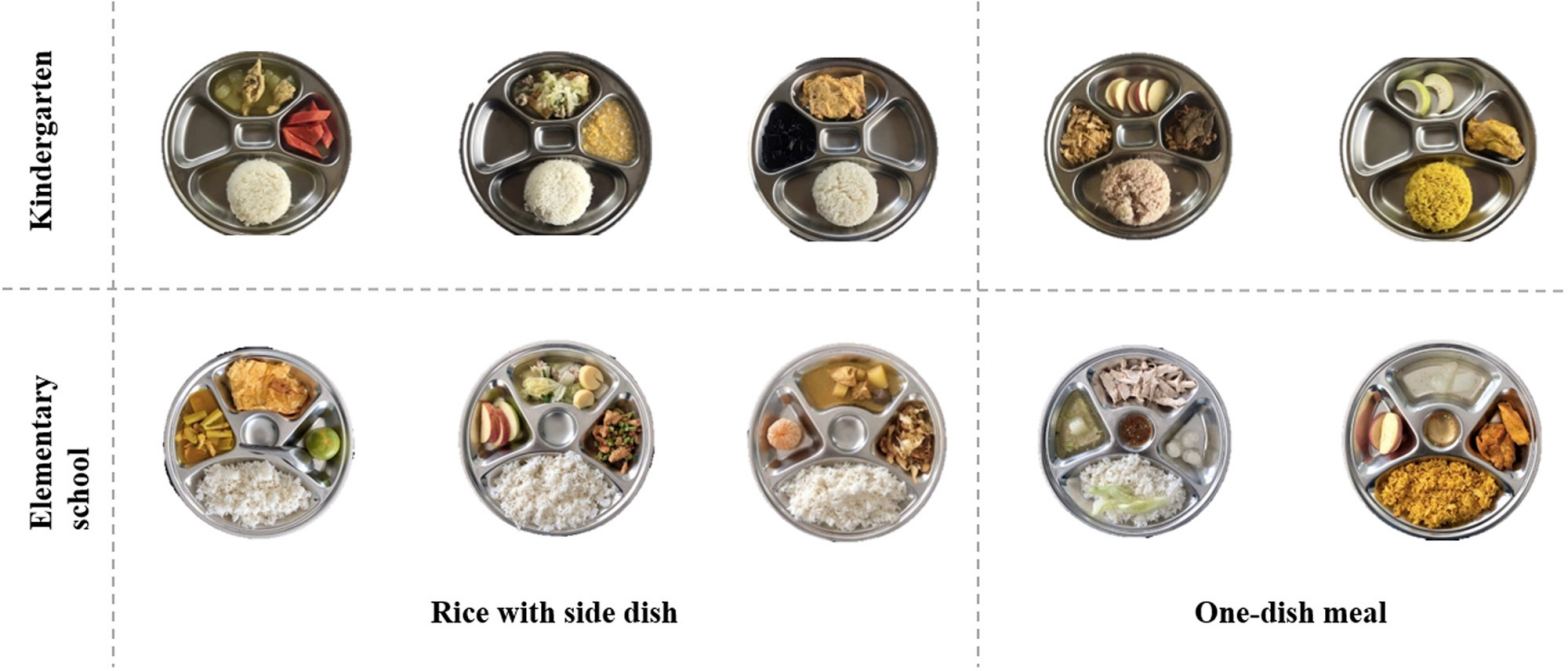
Lunch meals in school lunch programme during the assessment.

As mentioned earlier, three menus of rice with side dish and two menus of one-dish meal were served as lunch in each school during the 5-d assessment period (Fig. 1). Menus, appearance, main ingredients and nutritional composition of all meals are compiled in Supplementary Table S2. We observed that most food items in lunch menus of kindergartners were sliced, chopped, minced, except the drumsticks served with chicken biryani. Moreover, all the kindergarten menus had blander taste and less spiciness, comparing to those served at elementary school. Regarding the methods of cooking and preparation, lunch menus of both schools were cooked by boiling, streaming, and stir-frying. None of the menus was deep-fried food. Food items of each food group in the served lunch included (1) rice, i.e., steamed white rice, biryani rice, chicken-fat rice, shrimp paste-seasoned rice; (2) fruits, i.e., fresh-cut apple, guava, ripe papaya, tangerine, longan; (3) vegetables, i.e., cooked wax gourd, Chinese cabbage, sweet corn, string bean, morning glory, cabbage, green papaya and raw cucumber; (4) meats, i.e., pork, minced pork, caramelised pork, chicken; and (5) eggs, i.e., omelette, egg tofu.

The portion size of lunch served in kindergarten was 294 g for rice with side dish and 291 g for the one-dish meal. Meanwhile, the portion sizes of rice with side dish served at elementary school were 373 g, which was larger than 281 g for the one-dish meal, as well as than the same dish style served at kindergarten (Table 1). The amount per serving portion of each food group in the lunches served at kindergarten and elementary school is presented in Table 1. There was one rice with side dish menu of kindergarten that consisted only of rice and egg (plain omelette) without other food groups, while the other two menus of rice with side dish did not contain egg. For elementary school, a rice with side dish menu was lack of egg. Interestingly, vegetables and egg were missing from all one-dish meal menus of kindergarten and elementary school, respectively. In all cases, rice was the food group with the greatest amount, accounting for about 50–70 % of the portion weight. When the amounts of each food group in rice with side dish and in one-dish meal were compared, it was found that rice with side dish served at both schools contained less meats, but more vegetables, fruits and eggs. Rice with side dish menus served at kindergarten contained smaller amounts of all food groups, except eggs, than those served at elementary school. For one-dish meal, kindergarten pupils received more rice, fruits and eggs, but less meats and vegetables than elementary school students. Comparing with the recommended values for each age group in the Lunch Standard for Thai Children^(9)^, rice served in all menus at kindergarten was about doubled the recommended amount of 82⋅5 g, while that served for elementary students was 1⋅5 times exceeded the recommended amount of 137⋅5 g. Meats were presented at the amount lower than recommendation in most cases, except for one-dish meal of kindergarten. Vegetables and fruits served as lunch at both schools did not reach the recommended amount. Eggs in lunch menus of both schools were closed to or exceeded the recommendation, except for one-dish meal of elementary school.

**Table 1.** Composition of lunch meals in school lunch programme

Food groups	Kindergarten		Elementary school	
	Rice with side dish^a^	One-dish meal^b^	Rice with side dish^a^	One-dish meal^b^
Amount per serving portion				
Food served (g)	294⋅3 ± 27⋅8^e^	290⋅6 ± 12⋅5	373⋅4 ± 26⋅5^d^	281⋅1 ± 23⋅5
Rice (g)	157⋅9 ± 38⋅2^e^	176⋅4 ± 0⋅6	205⋅9 ± 8⋅4^d^	169⋅6 ± 13⋅1
Meats (g)	28⋅0 ± 30⋅0^d^^,^^e^	63⋅7 ± 13⋅9^e^	38⋅6 ± 18⋅5^d^	52⋅5 ± 8⋅8
Vegetables (g)	26⋅3 ± 23⋅0^d^^,^^e^	0⋅0 ± 0⋅0^e^	47⋅5 ± 8⋅1^d^	29⋅8 ± 7⋅9
Fruits (g)	48⋅5 ± 42⋅7^d^^,^^e^	33⋅8 ± 3⋅4^e^	43⋅5 ± 4⋅8^d^	29⋅3 ± 9⋅5
Eggs (g)	33⋅6 ± 31⋅1^d^	16⋅7 ± 23⋅6^e^	37⋅8 ± 43⋅3	0⋅0 ± 0⋅0
% Recommendation^c^				
Rice	191⋅4 ± 72⋅9^e^	213⋅8 ± 65⋅1^e^	149⋅7 ± 30⋅7^d^	123⋅3 ± 13⋅8
Meats	58⋅3 ± 53⋅7^d^	132⋅6 ± 31⋅2^e^	53⋅7 ± 23⋅5^d^	72⋅9 ± 13⋅3
Vegetables	75⋅5 ± 58⋅0^d^	0⋅0 ± 0⋅0^e^	67⋅9 ± 15⋅9^d^	42⋅6 ± 9⋅6
Fruits	71⋅9 ± 54⋅0^d^^,^^e^	50⋅1 ± 11⋅2^e^	32⋅3 ± 4⋅1^d^	21⋅7 ± 5⋅7
Eggs	167⋅8 ± 136⋅4^d^	83⋅5 ± 90⋅8^e^	189⋅2 ± 188⋅4^d^	0⋅0 ± 0⋅0

a Means ± standard deviations of ten portions of three menus.

b Means ± standard deviations of ten portions of two menus.

c Recommended amount for respective age group according to the Lunch Standard for Thai Children.

d Mean values of rice with side dish and one-dish meal of the same school are significantly different (*P* < 0⋅05).

e Mean values of the same dish style of kindergarten and elementary school are significantly different (*P* < 0⋅05).

Considering the macronutrients of lunch meals, rice with side dish menus served at kindergarten had lower carbohydrate, protein and energy than one-dish meals, while the fat content was not different (Table 2). An opposite trend was observed for menus of elementary school of which rice with side dish menus contained more carbohydrate and fat than one-dish meal, but the protein and energy contents were identical. When comparing a similar type of main dish, it was found that rice with side dish menus of kindergarten had lower carbohydrate, protein and energy contents than those provided for elementary students. For one-dish meal, menus at kindergarten contained higher carbohydrate, protein, fat and energy than those of elementary schools. Regarding the recommendation, macronutrients and energy content of the lunches provided at kindergarten exceeded the recommendation, except the carbohydrate content of rice with side dish menus. In case of elementary school, nutrients of rice with side dish were closed to the recommendation (95–102 %), but one-dish meal menus did not meet the recommendation for all nutrients except protein. In general, most nutrients of lunch meals served at kindergarten were 1⋅1–2⋅3 times higher than the recommendation, while carbohydrate in rice with side dish could cover 88 % of the recommended value for children under five. The amounts of carbohydrate and energy in lunch meals served at elementary school were closed to the recommended values. Excess protein content (130 % of recommended amount) was observed in both dish styles provided for elementary students, but the fat content in one-dish meal only approached 60 % of the recommended amount.

**Table 2.** Macronutrients and energy of lunch meals in school lunch programme

Nutrients	Kindergarten		Elementary school	
	Rice with side dish^a^	One**-**dish meal^b^	Rice with side dish^a^	One**-**dish meal^b^
Amount per serving portion				
Carbohydrate (g)	48⋅4 ± 15⋅7^d^^,^^e^	72⋅6 ± 23⋅5^e^	68⋅8 ± 13⋅2^d^	59⋅6 ± 5⋅3
Protein (g)	15⋅5 ± 4⋅1^d^^,^^e^	25⋅2 ± 5⋅3^e^	18⋅8 ± 6⋅3	18⋅9 ± 2⋅1
Fat (g)	16⋅5 ± 7⋅1	15⋅1 ± 3⋅1^e^	13⋅4 ± 12⋅0	8⋅3 ± 1⋅6
Energy (kcal)	407⋅6 ± 105⋅5^d^^,^^e^	529⋅6 ± 137⋅4^e^	475⋅5 ± 145⋅1^d^	392⋅3 ± 32⋅1
% Recommendation^c^				
Carbohydrate	88⋅1 ± 28⋅5^d^	132⋅2 ± 42⋅7^e^	97⋅1 ± 18⋅7	84⋅2 ± 7⋅4
Protein	143⋅2 ± 37⋅8^d^	233⋅3 ± 48⋅8^e^	134⋅1 ± 44⋅9	135⋅2 ± 15⋅1
Fat	152⋅6 ± 65⋅5^e^	139⋅7 ± 28⋅5^e^	95⋅6 ± 85⋅5	59⋅6 ± 11⋅3
Energy	113⋅2 ± 29⋅3^d^	146⋅7 ± 38⋅2^e^	102⋅3 ± 31⋅2^d^	84⋅4 ± 6⋅9

a Means ± standard deviations of ten portions of three menus.

b Means ± standard deviations of ten portions of two menus.

c Recommended amount for respective age group according to the Lunch Standard for Thai Children.

d Mean values of rice with side dish and one-dish meal of the same school are significantly different (*P* < 0⋅05).

e Mean values of the same dish style of kindergarten and elementary school are significantly different (*P* < 0⋅05).

### Quantity and composition of plate waste

On each assessment day, food scraps from 108 and 48 plates were collected at the kindergarten and elementary school, respectively. So, for the entire 5-d assessment period, plate waste data was obtained from 324 plates of rice with side dish and 216 plates of one-dish meal served at kindergarten, as well as 144 plates of rice with side dish and 96 plates of one-dish meal served at elementary school. On average, kindergarten pupils produced double larger amount of plate waste than did elementary students, regardless of the type of main dish served at lunch (Table 3). The amount of rice with side dish menus discarded by students at both schools were significantly larger than one-dish meal. Plate waste in kindergarten was 86 g/portion when rice with side dish was served at lunch, and 50 g/portion for one-dish meal. Each elementary student discarded 38 and 22 g of rice with side dish, and of one-dish meal, respectively.

**Table 3.** Quantity and composition of plate waste in school lunch programme

Food groups	Kindergarten		Elementary school	
	Rice with side dish^a^	One**-**dish meal^b^	Rice with side dish^a^	One**-**dish meal^b^
Plate collected	324	216	144	96
Wasted amount per serving portion				
Plate waste (g)	85⋅6 ± 13⋅5^c^^,^^d^	50⋅0 ± 4⋅8^d^	38⋅4 ± 5⋅2^c^	21⋅9 ± 2⋅9
Rice (g)	50⋅2 ± 10⋅7^c^^,^^d^	28⋅9 ± 2⋅9^d^	14⋅5 ± 3⋅1	14⋅1 ± 0⋅8
Meats (g)	4⋅3 ± 5⋅3^c^	13⋅4 ± 1⋅6^d^	5⋅2 ± 2⋅8	4⋅9 ± 1⋅2
Vegetables (g)	10⋅8 ± 9⋅5	0⋅0 ± 0⋅0^d^	16⋅5 ± 2⋅2^c^	2⋅2 ± 1⋅7
Fruits (g)	13⋅3 ± 11⋅9	5⋅3 ± 2⋅4^d^	0⋅5 ± 0⋅9	0⋅7 ± 0⋅8
Eggs (g)	7⋅1 ± 6⋅6	2⋅3 ± 2⋅7	2⋅0 ± 2⋅8	0⋅0 ± 0⋅0
% Wasted				
Plate waste	29⋅0 ± 2⋅3^c^^,^^d^	17⋅2 ± 1⋅0^d^	10⋅3 ± 1⋅3	7⋅9 ± 1⋅6
Rice	32⋅6 ± 8⋅8^c^^,^^d^	16⋅4 ± 1⋅7^d^	7⋅0 ± 1⋅3	8⋅4 ± 1⋅0
Meats	9⋅5 ± 8⋅7	22⋅0 ± 6⋅4^d^	13⋅3 ± 2⋅3	9⋅8 ± 3⋅6
Vegetables	27⋅3 ± 23⋅7	0⋅0 ± 0⋅0^d^	35⋅4 ± 7⋅0^c^	6⋅7 ± 4⋅4
Fruits	18⋅8 ± 17⋅9	16⋅3 ± 8⋅4^d^	1⋅4 ± 2⋅5	1⋅9 ± 2⋅1
Eggs	14⋅1 ± 12⋅2	7⋅0 ± 8⋅0	2⋅9 ± 3⋅1	0⋅0 ± 0⋅0

a Means ± standard deviations of 3 aggregate samples of 108 portions for kindergarten or 48 portions for elementary school.

b Means ± standard deviations of 2 aggregate samples of 108 portions for kindergarten or 48 portions for elementary school.

c Mean values of rice with side dish and one-dish meal of the same school are significantly different (*P* < 0⋅05).

d Mean values of the same dish style of kindergarten and elementary school are significantly different (*P* < 0⋅05).

The amount per capita of plate waste for each food group is presented in Table 3. At kindergarten, rice was the food group wasted at the largest amount, regardless of the types of main dish. About 50 g of rice from each portion of rice with side dish was wasted, while the amount wasted from one-dish meals was smaller at 29 g/portion. On the other hand, rice with side dish menus produced less meat scraps than one-dish meals. For elementary school, more vegetables were discarded when rice with side dish menus were served, while there was no significant difference in the plate waste amount of other food groups. When plate waste generated from similar dish style were compared among the schools, it was found that kindergartners wasted more rice, meats, vegetables and fruits than elementary students did, especially when one-dish meals were served as school lunch.

Considering the proportion of plate waste to the amount being served, it was revealed that kindergarten produced more plate waste in school lunch programme than elementary school, and that rice with side dish menus produced more plate waste than one-dish meal menus (Table 3). The food group wasted at the highest proportion varied among different dish styles and schools. Rice was the most wasted food group from rice with side dish menus at kindergarten, while that observed at elementary school was vegetables. For one-dish meal, meats were left uneaten the most at both schools. About one-third of the served rice was discarded from rice with side dish lunch menus by the kindergarten pupils. On the days that one-dish meal menus were provided, about two times smaller proportion of rice was left uneaten. For other food groups, there was no difference in the proportion being left on plate among different dish styles of the lunch meals. At elementary school, less than 15 % of the serving amounts of rice, meats, fruits and eggs were wasted from rice with side dish menus and one-dish meals. However, 35 % of the vegetables in rice with side dish menus were discarded by elementary students, which was about five times more than that of one-dish meals. Comparing the plate waste of similar dish style of lunch meals, a larger proportion of rice was discarded from both dish styles of the kindergarten than that of the elementary school. Moreover, for one-dish meal menus, the proportions of wasted meats, vegetables and fruits at the kindergarten were also larger than elementary school.

### Macronutrients and caloric content of plate waste

In terms of nutritional composition, carbohydrate was the nutrient with the largest content in plate waste from any types of lunch menu at both schools, accounting for 13–25 g/100 g waste. Fat was presented in a smaller amount than other nutrients in all cases. Caloric content of plate waste, which was calculated from the macronutrient content, ranged between 107 and 189 kcal/g (Table 4). At kindergarten, plate waste of rice with side dish contained less protein, fat and energy than the leftover of one-dish meal. For elementary school, plate waste of rice with side dish had lower carbohydrate, protein and energy contents than that of one-dish meals. The nutrients in food scraps from rice with side dish were similar at both schools, but the leftover from one-dish meal at kindergarten contained a larger amount of macronutrients.

**Table 4.** Macronutrients and energy of plate meals in school lunch programme

Nutrients	Kindergarten		Elementary school	
	Rice with side dish^a^	One**-**dish meal^b^	Rice with side dish^a^	One**-**dish meal^b^
Carbohydrate (g**/**100 g waste)	20⋅1 ± 4⋅7	25⋅2 ± 0⋅2^d^	13⋅2 ± 4⋅3^c^	20⋅3 ± 0⋅1
Protein (g**/**100 g waste)	4⋅5 ± 0⋅9^c^	9⋅5 ± 1⋅0^d^	5⋅0 ± 1⋅1^c^	8⋅1 ± 0⋅1
Fat (g**/**100 g waste)	2⋅2 ± 1⋅5^c^	5⋅6 ± 0⋅2^d^	3⋅8 ± 0⋅9	4⋅2 ± 0⋅5
Energy (kcal**/**100 g waste)	117⋅9 ± 33⋅7^c^	188⋅7 ± 1⋅4^d^	106⋅7 ± 9⋅8^c^	151⋅5 ± 3⋅9

a Means ± standard deviations of duplicate analyses of 3 aggregate samples of 108 portions for kindergarten or 48 portions for elementary school.

b Means ± standard deviations of duplicate analyses of 2 aggregate samples of 108 portions for kindergarten or 48 portions for elementary school.

c Mean values of rice with side dish and one-dish meal of the same school are significantly different (*P* < 0⋅05).

d Mean values of the same dish style of kindergarten and elementary school are significantly different (*P* < 0⋅05).

### Impacts of plate waste

Plate waste directly affected the actual amounts of food consumed by the students at lunch (Table 5). The results revealed that, in all cases, meats, fruits and vegetables that the students received from school lunch meals could not meet the recommended amounts for their age group, given only 21–66 % of the recommendation. On the other hand, rice was consumed at 113–178 % of the recommended amount (Table 5). For eggs, students at both schools obtained exceeding amount from rice with side dish menus but inadequate amount from one-dish meal menus. Kindergartners obtained meats at an inadequate amount from one-dish meal lunch menus, while the amount obtained from rice with side dish menus was in an opposite trend. For elementary students, intake of meats from both main dish types was lower than the recommendation.

**Table 5.** Estimated food intake from lunch meals in school lunch programme

Food groups	Kindergarten		Elementary school	
	Rice with side dish^a^	One**-**dish meal^b^	Rice with side dish^a^	One**-**dish meal^b^
Intake per capita				
Rice (g)	107⋅7 ± 34⋅8^d^^,^^e^	147⋅5 ± 3⋅4	191⋅4 ± 5⋅6^d^	155⋅5 ± 11⋅4
Meats (g)	23⋅6 ± 24⋅8	50⋅3 ± 13⋅0	33⋅4 ± 15⋅7	47⋅6 ± 8⋅4
Vegetables (g)	15⋅5 ± 13⋅5	0⋅0 ± 0⋅0^e^	31⋅0 ± 7⋅8	27⋅6 ± 4⋅7
Fruits (g)	35⋅2 ± 32⋅4	28⋅5 ± 5⋅2	43⋅0 ± 5⋅6	28⋅7 ± 7⋅0
Eggs (g)	26⋅5 ± 24⋅5	14⋅4 ± 16⋅6	35⋅8 ± 40⋅5	0⋅0 ± 0⋅0
% Recommendation^c^				
Rice	130⋅6 ± 42⋅2^d^	178⋅8 ± 4⋅1^e^	139⋅2 ± 4⋅1^d^	113⋅1 ± 8⋅3
Meats	49⋅2 ± 51⋅6	104⋅6 ± 27⋅0^e^	46⋅4 ± 21⋅9	66⋅0 ± 11⋅7
Vegetables	44⋅4 ± 38⋅7	0⋅0 ± 0⋅0^e^	44⋅3 ± 11⋅1	39⋅5 ± 6⋅8
Fruits	52⋅2 ± 48⋅1	42⋅2 ± 7⋅7^e^	31⋅8 ± 4⋅2^d^	21⋅2 ± 5⋅2
Eggs	132⋅4 ± 122⋅7	72⋅0 ± 83⋅1	179⋅2 ± 202⋅6	0⋅0 ± 0⋅0

a Means ± standard deviations of 3 aggregate samples of 108 portions for kindergarten or 48 portions for elementary school.

b Means ± standard deviations of 2 aggregate samples of 108 portions for kindergarten or 48 portions for elementary school.

c Recommended amount for respective age group according to the Lunch Standard for Thai Children.

d Mean values of rice with side dish and one-dish meal of the same school are significantly different (*P* < 0⋅05).

e Mean values of the same dish style of kindergarten and elementary school are significantly different (*P* < 0⋅05).

The impacts of plate waste in school lunch programme of both schools were assessed in terms of losses in quantity, calories and money (Table 6). The average plate waste of kindergartner was 71 g/capita/d. It was estimated that the entire kindergarten (531 students) would generate about 38 kg of food waste daily or about 7⋅5 metric tons in each school year. Such plate waste resulted in the caloric loss of 146 kcal per capita or 77 600 kcal on every school day. The amount of food discarded daily by each kindergarten student costed about 5 Thai baht or 0⋅16 US dollar. The estimated daily cost of uneaten lunch at kindergarten was about 2600 Thai baht (86 US dollars), from which the monetary cost in each school year would be as much as 500 000 Thai baht (16 000 US dollars). At elementary school, the average amount of plate waste was about 32 g/capita/d. By estimation, the school lunch programme would contribute to 7⋅6 kg of food waste for each school day, or 1⋅5 metric tons for each school year. The daily caloric loss from plate waste was 125 kcal per elementary pupil, or 29 800 kcal/d for the entire school of 239 students. That plate waste amount was equivalent to the monetary loss of 2 Thai baht per capita (0⋅07 US dollar), or 450 Thai baht (15 US dollars) on each day of school lunch programme implementation, which would sum up to about 90 000 Thai baht (3000 US dollar) for each school year.

**Table 6.** Estimated impacts of plate waste in school lunch programme

Impacts	Kindergarten (531 students)			Elementary school (239 students)		
	Capita^a^	School day^b^	School year^c^	Capita^a^	School day^b^	School year^c^
Quantity loss (g)	71⋅4 ± 21⋅9	37⋅9 × 10^3^	7582 × 10^3^	31⋅8 ± 10⋅0	7⋅6 × 10^3^	1520 × 10^3^
Caloric loss (kcal)	146⋅2 ± 45⋅5	77 632⋅2	15 526 440	124⋅7 ± 25⋅5	29 803.3	5 960 660
Financial loss (Thai baht)	4⋅8 ± 1⋅3	2548⋅8	509 760	1⋅9 ± 0⋅4	454⋅1	90 820

a Means ± standard deviations of 5 aggregate samples of 108 portions for kindergarten or 48 portions for elementary school.

b Calculated by multiplying per capita values with the number of students.

c Calculated by multiplying per school day values with the number of school day in a school year (200).

## Discussion

In the present study, lunch provided at kindergarten and elementary schools were varied in menu, portion size and nutritional quality. It was obvious that larger portions of rice with side dish were served for elementary students (Supplementary Table S2), despite the smaller recommended amount for each food group. According to the Lunch Standard for Thai Children, rice, vegetables and fruits should be included daily. Fishes, meats and eggs, which are sources of proteins, are recommended twice a week each, together with one day each of liver and tofu^(9)^. Although meal arrangement was performed using online platform according to the guideline, none of the menus served during the assessment period contained fish, liver and tofu (Supplementary Table S2). Moreover, some menus were lack of one or more of the five food groups considered in this survey (Table 1). All the menus served during the assessment period had inadequate fruits and vegetables, while containing the excess amount of rice. It could be that food items in the menus obtained from calculation might be modified by the teachers or cooking staff, aiming to match the food preference or eating habit of the students, especially kindergartners. Nutrient contents in lunch portions of kindergarten were more deviate from the recommended values than those of elementary school (Table 2), which was consistent with the bigger portion size and amount of rice at the kindergarten (Table 1). In addition, nutritional quality of rice with side dish menus was more balanced than one-dish meals, probably because they contained more food groups.

To our best knowledge, this is the first study on food waste in school lunch programme in Thailand. In total, the plate waste of 780 portions of school lunch was assessed in the present study. The greater amount of plate waste generated by each kindergartner (Table 3) could be explained by the fact that the portion size of lunch served for them was even larger than for elementary students (Supplementary Table S2). Kindergartners are younger than elementary students and thus they tended to eat less. As informed by teachers during informal interviews, early-year students were likely to reject school lunch. This is due to their unfamiliarity with school dining and the food provided as school lunch. Consequently, kindergartners wasted a larger proportion of nutrients available in their school lunch than did the elementary students. Another possible reason for kindergarteners to discard their lunch could be the appropriateness of menus and the food items. Younger children at kindergarten would prefer having meals with less flavourful taste, softer texture or smaller pieces than the elementary students. This was consistent with the characteristics of the lunch menus served at both schools (Supplementary Table S2).

One-dish meal menus seemed to be more accepted by the students, comparing with rice with side dish, and hence they were less discarded (Table 3). It was shown that poor food quality or presentation, as well as inappropriate lunch time and too-short lunch break period could be the causes of plate waste in schools^(32,33)^. Based on our observation, one-dish meal menus at both schools were served in a more eye-appealing manner than rice with side dish. It is well documented that food preference and sensory quality are the two first deciding factors for food purchasing and consumption of Thai children aged 6–14 years^(34)^. Furthermore, children, particularly kindergartners, naturally stop eating when they have had enough, without being aware of wasting the food. It is suggested that portion size and sensory quality, especially appearance, texture and taste must be more considered for menu setting in school lunch programme. Within a meal, there should be a pleasing combination of different sizes and shapes of foods, such as cubes, shredded bits and strips. The arrangement of menu items on the serving tray could also enhance the attractiveness of the meal to young children^(35)^. Moreover, palatability of meals can be enhanced if they are served in a pleasant and comfortable setting. Therefore, the government or responsible agencies should provide regular training for schoolteachers and school cooks on menu designing for better planning and setting the menus according to the school lunch standards. In addition, conducting the survey on students’ food satisfaction and preference, as well as running the campaigns to improve food waste awareness among the students would help in reducing the plate waste^(36)^. Several studies have shown that interventions, such as information technology, policy, system and practice change, were efficient in reducing food waste at the consumption stage^(37)^. Practice change could increase the amount of entrée and main dishes consumed by students in US middle schools and Portuguese public primary schools^(38,39)^. Serving smaller portions of meals or staple food has been proven to reduce plate waste generated by university students in The Netherlands and Thailand^(40,41)^.

The amount per capita of plate waste obtained from the present study was lower than food waste generated per capita report at 58 g/portion in the Finnish food service sector^(5)^, except for the rice with side dish at kindergarten. The percentage of food being discarded or food waste index can be used as the indicator of the amount of plate waste generated^(17)^. Up to 30 % of the lunch served at kindergarten became food waste (Table 3), which was classified elsewhere as ‘Unacceptable’^(42)^. For elementary school, 8–10 % of the lunch served was discarded, which fitted in a better rating, ‘Bad’. Such numbers were comparable to that reported for schools in the Beijing metropolitan of China (21 %)^(21)^. However, the lower amount of plate waste was reported in previous studies conducted in canteens in Finland (5⋅7 %) and Italy (15 %)^(5,43)^. The composition of plate waste at both schools in the present study depended on the initial serving amount of each food group. Rice was wasted the most because the amount served was two times larger than the recommended amount (Tables 1 and 3). Therefore, carbohydrates became the major nutrient in the plate waste (Table 4). On the other hand, food groups that the amount on plates was small or short of the recommendation were less discarded, except for vegetables (Tables 1 and 3). This indicated the low preference of students for vegetables. A previous study in low-income schools in Texas, USA also found that younger students discarded significantly more food of all groups than older students, which accounted for greater losses in nutrients and calories^(44)^. In addition, the proportion of food items being discarded from school lunch meals in the present study was lower than that of the US schools. A study in the cafeteria of a US school revealed that 51 % of vegetables, 51 % of entrées, 45 % of milk and 33 % of fruits were wasted by pre-kindergartners and kindergartners^(45)^. In addition, in a plate waste study conducted in schools located in Massachusetts, USA, students of grades 3 through 8 threw away 12 % of their meals, 46 % of milk, 59 % of vegetables and 45 % of fruits^(4)^.

Plate waste affected the amount of food and nutrients that students would benefit from having nutritious food at school. In the present study, the amount of each food group and nutrient consumed by students depended on the type of main dish and the school (Table 5). This was due to the different amounts of food and nutrient in the provided lunch portions. In most cases, the amounts of rice, and eggs that kindergarten and elementary students actually consumed at lunch, were higher than recommendation from the Thai school lunch programme^(9)^, but the amounts of meats, fruits and vegetables consumed were much below the standard, except for one-dish meal menus of the kindergarten (Table 5). The findings were in line with observations on students in US elementary schools that younger students consumed significantly less vegetables, whole grains and total protein than the older ones^(46)^. The amounts of food and nutrient received from school lunch were superior to the consumption pattern reported for Thai children. A survey of the National Statistical Office of Thailand reported that meat and poultry, eggs, and fruits and vegetables were consumed daily by only 10, 25 and 33 % of the population group of 6–14 years, respectively^(34)^. It was demonstrated that the majority of Thai children aged 0⋅5–12 years consumed less energy than the recommendation^(2)^. A plate waste study in US elementary schools also revealed that amounts of fruit, vegetables, whole grains, protein foods and milk consumed by students of all grade levels were in accordance with the menu pattern of its National School Lunch Programme^(46)^. It could be inferred that students at both schools would receive the excess amount of carbohydrate from their lunch meals, while the protein intake would below the recommendations. Considering the nutrient loss associated with plate waste, it has been reported that the caloric values of plate waste of students in Poland were minimal, with a median caloric content of below 15 kcal per capita^(47)^. Such value was much lower than the calories of plate waste per capita in our study that up to 146 kcal loss per capita was observed (Table 6). The differences between values obtained from the present study and those previously reported were due to the variations in food characteristics, food serving styles, consumption patterns, as well as waste quantification methodologies.

Impacts of plate waste in school lunch programme varied with the quantity of plate waste and the number of students (Table 6). In the present study, there were more students at kindergarten than elementary school (531 and 239 students, respectively). The daily amount of plate waste in kindergarten was equivalent to 126 lunch portions (about 300 g each) being served, which could cover 23 % of the kindergartners. The nutrients discarded with plate waste on each day were enough to meet recommended daily caloric intake of 44 kindergartners aged 3–5 years (1200 kcal/d)^(9)^. The monetary loss due to plate waste was equivalent to the daily budget of school lunch allocated for 129 students or 24 % of the entire kindergarten. It could infer that by cutting down 50 % of plate waste, such school would save about 250 000 Thai baht (8000 US dollars) of its annual budget for school feeding. The impacts of plate waste were less significant at the elementary school. The quantity of plate waste on each day was comparable to seven servings of lunch. Such plate waste contained caloric content that was adequate to fulfil the daily requirement of five children aged 6–12 years (1550 kcal/d)^(9)^. The daily cost of plate waste was enough to cover the lunch budget for twenty-two students or about 10 % of the students at elementary school. There would also be other hidden socioeconomic and environmental costs associated with plate waste in school lunch programme. Transportation, energy and labour costs were also excluded from the cost of wasted food calculations^(48)^. Therefore, strategies on reduction of plate waste in school feeding should not only consider children's preferences and nutritional quality, but the actual impacts from nutritional, economic and environmental standpoints should also be addressed in such effort^(49)^. The Costa Rican food loss and waste reduction network indicated that collaborative actions among institutions and sectors are vital in promoting food loss and waste reduction^(50)^. It should be noted that the management of plate waste and food waste did not exist in both schools of the present study. Food waste was compiled with other garbage prior to being collected and handled by the local administration. Together with organic waste, food waste is managed by composting and landfilling, which contributes to the greenhouse gases that cause global warming and the climate crisis^(51)^.

The key strength of the present study is that it provides the first assessment of plate waste and nutrient loss due to plate waste in school lunch programme of Thailand. The data offered a detailed picture of nutrient loss, including percentages of loss by food group. Comparisons were also made with the standard for school lunch, which provided the basic information on the nutritional quality of lunch. However, the fact that the survey was conducted only in two schools would be the limitation of this cross-sectional study. Both schools are medium-sized with 121–600 students, according to the classification of Thailand's Ministry of Education. Such school size was the majority (70 %) of schools in Thailand^(25)^. Ratios of male and female students were about 1:1 for both schools. The distribution of weight-for-height of students at both schools was in accordance with the national-level scenario^(1)^. The district where the study was conducted is categorised, based on its population size, as a semi-urban area. The average household annual income is comparable to that of semi-urban areas in other provinces, and the vicinity areas of the capital city of Thailand^(31)^. Therefore, it was plausible that the two cases in the present study could be generalised to provide a rough perspective of the situation of plate waste school lunch programme in Thailand. Further studies should be conducted at more schools countrywide to obtain a more representing data on school food waste.

## Conclusion

The present study provided information on the amount and composition of plate waste in school lunch programme, as well as its nutritional and financial impacts. Overall, composition and nutritional quality of lunch meals served at schools tended to deviate from the adopted national standards. Plate waste depended largely on the portion size and composition of the provided lunch. Consequently, students’ food consumption levels were below the recommended amounts for school lunch, particularly of meats, vegetables and fruits. The caloric and costs associated with discarded foods were evident. Capacity building and training of teachers and staff involving in school lunch programme should be provided to improve the adequacy of food and nutrient, and nutritional quality of the school lunch. Such actions will facilitate the reduction of plate waste in school lunch programme. The findings of the present study should be a good foundation for future research of its kind, and useful preliminary data for efficiently designing and implementation of sustainable school lunch programme in Thailand and other middle-income countries with similar context.

## Supplementary material

For supplementary material accompanying this paper visit https://doi.org/10.1017/jns.2022.31. click here to view supplementary material
