# Nutritional quality of lunch meals and plate waste in school lunch programme in Southern Thailand — CORRIGENDUM

**DOI:** 10.1017/jns.2023.56

**Published:** 2023-06-23

**Authors:** Jaruneth Petchoo, Narisara Kaewchutima, Nattapol Tangsuphoom

Details: Correct approval number for human research ethics in ‘Methods’ section

Currently reads: WUEC-19-173-01

This should read: WUEC-20-073-01

Typographical error of the approval number for human research ethics (WUEC-19-173-01), as appeared in Methods part on the 2nd page of the published version. The correct approval number is WUEC-20-073-01

