# Supplementary material for: Nutritional quality of lunch meals and plate waste in school lunch programme in Southern Thailand
Source: J Nutr Sci. 2022 May 12;11:e35. doi: 10.1017/jns.2022.31 (PMC9108001; doi:10.1017/jns.2022.31)
Supplement: Supplementary file 1 [file S2048679022000313sup001.docx]

**SUPPLEMENTAL MATERIALS**

**Table S1.** Anthropometric indices of students

| **Anthropometric indices** | **Kindergarten**  (age 4–6 years) | | **Elementary school**  (age 7–12 years) | |
| --- | --- | --- | --- | --- |
|  | **N** | **%** | **N** | **%** |
| **Gender** |  |  |  |  |
| Boys | 257 | 48.4 | 127 | 53.1 |
| Girls | 274 | 51.6 | 112 | 46.9 |
| **Weight for age** |  |  |  |  |
| <-2 S.D. | 15 | 2.8 | 117 | 48.9 |
| -1.5 S.D. to -2 S.D. | 24 | 4.5 | 14 | 5.9 |
| -1.5 S.D. to +1.5 S.D. (Median) | 454 | 85.5 | 88 | 36.8 |
| +1.5 S.D. to +2 S.D. | 12 | 2.3 | 3 | 1.3 |
| >+2 S.D. | 26 | 4.9 | 17 | 7.1 |
| **Height for age** |  |  |  |  |
| <-2 S.D. | 10 | 1.9 | 142 | 59.4 |
| -1.5 S.D. to -2 S.D. | 17 | 3.2 | 7 | 2.9 |
| -1.5 S.D. to +1.5 S.D. (Median) | 462 | 87.0 | 80 | 33.5 |
| +1.5 S.D. to +2 S.D. | 24 | 4.5 | 6 | 2.5 |
| >+2 S.D. | 18 | 3.4 | 4 | 1.7 |
| **Weight for height** |  |  |  |  |
| <-2 S.D. (Extreme underweight) | 13 | 2.4 | 5 | 2.1 |
| -1.5 S.D. to -2 S.D. (Underweight) | 26 | 4.9 | 16 | 6.7 |
| -1.5 S.D. to +1.5 S.D. (Normal) | 435 | 81.9 | 166 | 69.4 |
| +1.5 S.D. to +2 S.D. (Overweight) | 7 | 1.3 | 15 | 6.3 |
| +2 S.D. to +3 S.D. (Obesity) | 21 | 4.0 | 17 | 7.1 |
| >+3 S.D. (Extreme obesity) | 29 | 5.5 | 20 | 8.4 |

**Table S2.** Menus and nutritional composition of main ingredients served in school lunch program

| **Name of Menu** | **Food item/ ingredient** | | | **Amount^†^ (g/portion)** | **Nutritional composition^‡^** | | | | | | |
| --- | --- | --- | --- | --- | --- | --- | --- | --- | --- | --- | --- |
|  |  |  |  |  | **Carbo**  **hydrate (g)** | | **Protein**  **(g)** | | **Fat**  **(g)** | **Energy (kcal)** | |
| **Kindergarten** | | | | | | | | | | | |
| Rice with side dish: Steamed white rice, chicken and wax gourd soup, papaya | | | | | | | | | | | |
| 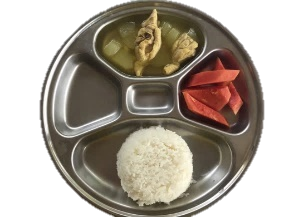 | White rice, cooked | | | 144.6 | 45.0 | | 3.3 | | 0.3 | 195.9 | |
|  | Chicken thigh, cooked | | | 59.6 | 0.0 | | 14.0 | | 7.4 | 122.6 | |
|  | Wax gourd | | | 42.5 | 1.1 | | 0.2 | | 0.0 | 5.2 | |
|  | Papaya, ripe | | | 65.0 | 4.8 | | 0.4 | | 0.1 | 21.7 | |
|  | Total | | | 311.7 | 50.9 | | 17.9 | | 7.8 | 345.4 | |
| Rice with side dish: Steamed white rice, Chinese cabbage soup with minced pork and omelet, sweet corn gruel with coconut milk | | | | | | | | | | | |
| 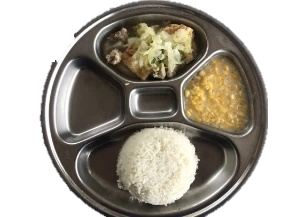 | White rice, cooked | | | 128.1 | 39.8 | | 2.9 | | 0.3 | 173.5 | |
|  | Minced pork, cooked | | | 24.3 | 0.0 | | 5.9 | | 3.6 | 56.0 | |
|  | Omelet | | | 39.3 | 0.9 | | 5.5 | | 12.9 | 141.7 | |
|  | Chinese cabbage | | | 36.8 | 0.3 | | 0.3 | | 0.0 | 2.4 | |
|  | Sweet corn | | | 80.5 | 8.4 | | 2.3 | | 4.3 | 81.5 | |
|  | Total | | | 309.0 | 49.4 | | 16.9 | | 21.1 | 455.1 | |
| Rice with side dish: Steamed white rice, omelet, grass jelly in syrup | | | | | | | | | | | |
| 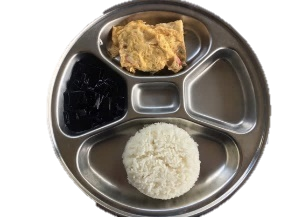 | White rice, cooked | | | 130.0 | 40.4 | | 3.0 | | 0.3 | 176.3 | |
|  | Omelet | | | 61.4 | 1.5 | | 8.6 | | 20.2 | 222.2 | |
|  | Grass jelly | | | 70.9 | 2.8 | | 0.1 | | 0.0 | 11.6 | |
|  | Total | | | 262.3 | 44.7 | | 11.7 | | 20.5 | 410.1 | |
| One-dish meal: Shrimp paste-seasoned rice (seasoned rice, caramelized pork, omelet), apple | | | | | | | | | | | |
| 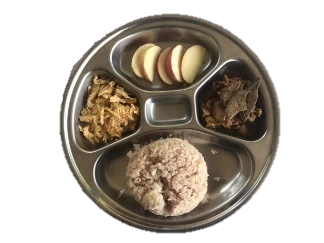 | Shrimp paste-seasoned rice | | | 176.0 | 54.7 | | 4.0 | | 0.4 | 238.4 | |
|  | Caramelized pork | | | 53.8 | 23.6 | | 18.3 | | 4.1 | 204.5 | |
|  | Omelet | | | 33.4 | 0.8 | | 4.7 | | 11.0 | 121.0 | |
|  | Apple | | | 36.2 | 4.5 | | 0.1 | | 0.0 | 18.4 | |
|  | Total | | | 299.4 | 83.6 | | 27.1 | | 15.5 | 582.3 | |
| One-dish meal: Chicken biryani, guava | | | | | | | | | | | |
| 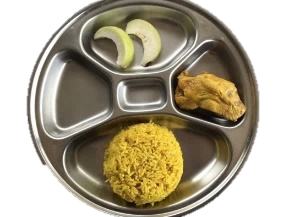 | Biryani rice | | | 176.8 | 59.3 | | 5.9 | | 5.5 | 310.3 | |
|  | Chicken thigh, cooked | | | 73.5 | 0.0 | | 17.3 | | 9.1 | 151.1 | |
|  | Guava | | | 31.4 | 2.2 | | 0.2 | | 0.0 | 9.6 | |
|  | Total | | | 281.7 | 61.5 | | 23.4 | | 14.6 | 471.0 | |
| **Elementary school** | | | | | | | | | | | |
| Rice with side dish: Steamed white rice, green papaya and morning glory sour soup with pork, omelet, tangerine | | | | | | | | | | | |
| 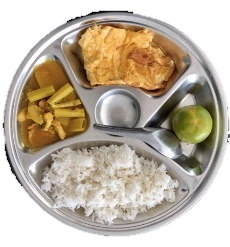 | | White rice, cooked | 196.2 | | | 61.0 | 4.5 | 0.5 | | | 266.5 |
|  |  | Pork, cooked | 24.0 | | | 0.0 | 8.0 | 0.5 | | | 36.5 |
|  |  | Omelet | 85.0 | | | 2.0 | 11.9 | 28.0 | | | 307.6 |
|  |  | Papaya, green | 20.7 | | | 0.6 | 0.1 | 0.0 | | | 2.8 |
|  |  | Morning glory | 17.6 | | | 0.1 | 0.3 | 0.1 | | | 2.5 |
|  |  | Tangerine | 43.7 | | | 3.6 | 0.4 | 0.1 | | | 16.9 |
|  |  | Total | 387.2 | | | 67.3 | 25.2 | 29.2 | | | 632.8 |
| Rice with side dish: Steamed white rice, cabbage soup with egg tofu and minced pork, spicy stir-fried chicken with holy basil and string bean, apple | | | | | | | | | | | |
| 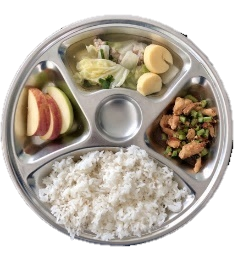 | White rice, cooked | | | 210.2 | 65.4 | | 4.8 | | 0.5 | 285.3 | |
|  | Pork, cooked | | | 22.8 | 0.0 | | 5.6 | | 3.4 | 53.0 | |
|  | Chicken thigh, cooked | | | 36.6 | 0.0 | | 7.2 | | 3.0 | 55.8 | |
|  | Egg tofu | | | 28.5 | 0.5 | | 1.3 | | 0.9 | 15.3 | |
|  | Chinese cabbage | | | 37.6 | 0.3 | | 0.3 | | 0.0 | 2.4 | |
|  | String bean | | | 15.7 | 0.4 | | 0.4 | | 0.0 | 3.2 | |
|  | Apple | | | 38.7 | 4.8 | | 0.1 | | 0.0 | 19.6 | |
|  | Total | | | 390.1 | 71.4 | | 19.7 | | 7.8 | 434.6 | |
| Rice with side dish: Steamed white rice, chicken curry with wax gourd, stir-fried mung bean glass noodle with cabbage and chicken, tangerine | | | | | | | | | | | |
| 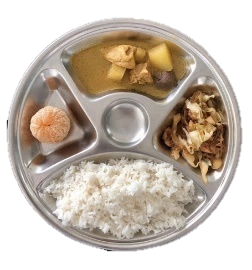 | White rice, cooked | | | 179.5 | 55.8 | | 4.1 | | 0.4 | 243.2 | |
|  | Mung bean glass noodle | | | 31.8 | 6.4 | | 0.0 | | 0.0 | 25.6 | |
|  | Chicken thigh, cooked | | | 32.5 | 0.0 | | 6.4 | | 2.7 | 49.9 | |
|  | Wax gourd | | | 26.1 | 0.7 | | 0.1 | | 0.0 | 3.2 | |
|  | Cabbage | | | 24.8 | 0.8 | | 0.4 | | 0.0 | 4.8 | |
|  | Tangerine | | | 48.2 | 3.9 | | 0.4 | | 0.1 | 18.1 | |
|  | Total | | | 342.9 | 67.6 | | 11.4 | | 3.2 | 344.8 | |
| One-dish meal: Chicken biryani and wax gourd soup, apple | | | | | | | | | | | |
| 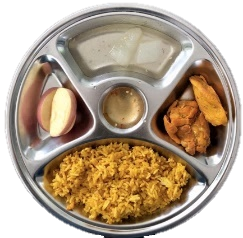 | Biryani rice | | | 171.8 | 53.4 | | 3.9 | | 0.4 | 232.8 | |
|  | Chicken thigh, cooked | | | 58.2 | 0.0 | | 14.8 | | 6.5 | 117.7 | |
|  | Wax gourd | | | 24.2 | 0.6 | | 0.1 | | 0.0 | 2.8 | |
|  | Apple | | | 36.0 | 4.4 | | 0.1 | | 0.0 | 18.0 | |
|  | Total | | | 297.7 | 58.4 | | 18.9 | | 6.9 | 371.3 | |
| One-dish meal: Chicken rice and wax gourd soup, longan | | | | | | | | | | | |
| 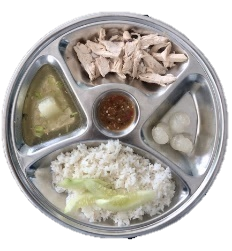 | Chicken-fat rice | | | 160.3 | 53.8 | | 4.9 | | 5.6 | 285.2 | |
|  | Chicken thigh, cooked | | | 46.2 | 0.0 | | 13.2 | | 4.0 | 88.8 | |
|  | Wax gourd | | | 13.5 | 0.4 | | 0.1 | | 0.0 | 2.0 | |
|  | Cucumber | | | 21.9 | 0.7 | | 0.2 | | 0.0 | 3.6 | |
|  | Longan | | | 22.6 | 3.6 | | 0.3 | | 0.0 | 15.6 | |
|  | Total | | | 264.5 | 58.5 | | 18.7 | | 9.6 | 395.2 | |

^†^Mean of 10 portions served; ^‡^Calculated using data from Online Thai Food Composition Database 2015
